# Quality of life of patients with traumatic spinal cord injuries: a cross-sectional study at a tertiary hospital in Uganda

**DOI:** 10.4314/ahs.v23i3.60

**Published:** 2023-09

**Authors:** Rogers Mugisa, Edward Luke Kironde, Erisa Sabakaki Mwaka

**Affiliations:** 1 Department of Orthopaedics, College of Health Sciences Makerere University, Kampala; 2 Mulago National Referral Hospital, Kampala; 3 Department of Anatomy, College of Health Sciences, Makerere University, Kampala

**Keywords:** Quality of life, traumatic injury, spinal cord

## Abstract

**Background:**

The study aimed to assess the perceived quality of life of patients with traumatic spinal cord injuries.

**Methodology:**

This was a cross sectional study conducted in the Spine Unit of a tertiary hospital in Uganda. The study population comprised of patients with spinal cord injuries. Data were collected using the WHO Quality of Life Brief questionnaire and Functional Independence Measure tool.

**Results:**

103 patients participated in the study, most were male (73.8%), and had a mean age of 37.7 years. Most participants were married (57.3%), unemployed (72.8%) and had no steady source of income (62.1%). Road traffic accidents accounted for most injuries (59.2%). The mean duration since injury was 20.5 months. Most participants (58.3%) had incomplete spinal cord injuries and 84.5% had complications. The perceived overall quality of life was poor in 87.4% of patients. Being employed (p= 0.02), the presence of complications (p= 0.03), and injury severity (p= 0.003) significantly affected quality of life. Functional independent measure scores were significantly better in individuals less severe injuries and those with lumbar level of injury with mean scores of 113.1±8.9 and 99.9±15.3 respectively.

**Conclusion:**

The overall self-reported quality of life among patients with traumatic spinal cord injury was generally poor.

## Introduction

Low- and middle-income countries (LMICs) are disproportionately affected by Road traffic injuries (RTI), with over 93% of road traffic deaths occurring in LMICs, despite possessing only 60% of global vehicles 1. The economic burden attributed to these injuries may cost up to 10% of their yearly gross domestic product 1, 2. Over five million people die from consequences of trauma and injury worldwide annually, with millions more living with post-injury disabilities 3. Like many other developing countries, Uganda is experiencing a growing burden of traumatic orthopaedic injuries 4 primarily resulting from road traffic accidents, falls, occupational accidents, violence and gunshot wounds 5. It is estimated that 64% of injuries in the Uganda are due to road traffic accidents 6; among these are spinal cord injuries (SCI).

There is dearth of literature on traumatic spinal cord injuries from the African continent, most of the information available is from South Africa 7-12. Traumatic spinal cord injuries (TSCI) are very devastating and cause profound life changes in millions of people globally. The overall global incidence of TSCI has been estimated at 10.5 cases per 100,000 persons 4 and 26 cases per million per year in developing countries 13-15. Traumatic SCI results in disability of varying severity and has implications on quality of life (QOL), and certain personal and social factors can positively or negatively influence the life of these individuals 5, 16. According to WHO the concept of QOL, has been defined as “an individuals' perceptions of their position in life in the context of the culture and value systems in which they live and in relation to their goals, expectations, standards, and concerns” 4, 17, 18. This definition is multidimensional, encompassing physical health, psychological health, and level of independence, social relationships, and the environment 19. Measuring the QOL is important and improving patients' QOL has increasingly become an important part of therapeutic goals to help patients feel and function as normally as possible 17, 18. Improving patients' QOL has increasingly become an important part of therapeutic goals 20. Provision of a holistic package of care to patients with TSCI is key to their wellbeing. Assessment of the QOL in patients with SCI could help in guiding and improving care for the affected individuals, development of SCI care protocols, enhancement of psychosocial or psychiatric services, and influence policy decisions. The study thus set out to evaluate the QOL in patients living with TSCI receiving treatment at a national referral tertiary hospital.

## Methodology

This was a cross-sectional study conducted between November 2019 and February 2020 in a specialized Spine Unit at a national referral tertiary hospital in Uganda. The study population comprised of adult patients with TSCI sustained at least six months previously and had been discharged from hospital back to their communities and voluntarily provided written informed consent. Participants were either contacted by phone or directly approached during their routine follow up outpatient visits. Home visits were made to those who expressed willingness to participate but could not to travel to hospital. Participants were consecutively recruited. Consecutive sampling was used because there is no spine trauma registry in Uganda and the number of patients with TSCI in Uganda is unknown. Further, most of the patients are referred to peripheral hospital within the vicinity of their home districts for post-hospital follow-up, therefore we were not sure whether we would achieve our estimated sample size. We therefore used non-probability sampling in order for us to obtain the sample size in the stipulated period. Patients with altered cognition and those that had difficulty in communicating were excluded.

Data were collected using assisted questionnaires adapted from two tools. Quality of life was assessed using the abbreviated version of the World Health Organization QOL Brief questionnaire (WHO-QOL-BREF) 21. This is a self-report questionnaire containing 26 items rated on five- point Likert scales. The tool evaluates six domains of QOL including physical health, psychological health, social relationships, environment, health and overall self-perceived QOL. The level of activity or disability was assessed using the Functional Independence Measure (FIM) tool. The FIM is one of the most accepted and commonly used instruments designed to measure functioning in activities of daily living and indicates the severity of the disability in persons with SCI 22-24. The tool assesses self-care, sphincter control, mobility or transfers, locomotion, communication and social integration; and all together evaluate the performance of 18 tasks. All the tasks are ranked on an ordinal scale ranging from 1(total assist) to 7. A FIM item score of 7 (complete independence) is categorized as “complete independence”, a score of 1 is “total assist”. Scores below 6 require another person for assistance. The total score ranges from 18 to 126 24. The severity of injury was assessed using the American spinal injury association (ASIA) impairment scale 25. On average, questionnaire completion took between 45- 60 minutes.

### Data analysis

The overall QOL score was computed as the summation of the 0-100 transformed scores of each domain (26). Data were summarized using descriptive statistics. Analysis of variance (ANOVA) and the student t-test were used to test the relationship between QOL, and the predictor variables. Multiple linear regression analysis was used to determine predictors of QOL. All variables that had p-values ≤ 0.2 were included in multivariate analysis. Regression coefficients (β) and 95% confidence intervals (CI) were reported. The level of significance was set at p value of 0.05.

### Ethical considerations

The study was approved by Makerere University School of Medicine Research Ethics Committee (REF SOM-REC 2019-140). Written informed consent was obtained from all participants prior to enrolment in the study. All participants were assured of confidentiality. Participants received transport reimbursement and were compensated for their time

## Results

A total of 103 patients participated in the study, a majority of which was male (73.8%) with a mean age of 37.7 years (SD14.7, range 18- 80). Most participants were married (57.3%), unemployed (72.8%) and had no steady source of income (62.1%). Participant demographic and injury characteristics are summarized in Table 1.

**Table 1:** Participant demographic and injury characteristics

Characteristic			
(N= 103)	Freq (%)	Characteristic	Freq (%)
**Age (Years)**		**Mechanism of injury**	
18-27	32 (31.1)	Fall from a height	13 (12.6)
28-37	29 (28.2)	Road traffic crush	61 (59.2)
38-47	14 (13.6)	Others	29 (28.2)
48-57	16 (15.5)	**Presence of complications**	
58-67	9 (8.7)	Symptom absent	16 (15.5)
68-77	1 (1.0)	Symptom present	87 (84.5)
78-87	2 (1.9)	**Duration of Injury (Months)**	
		Mean(SD)	
**Sex**			20.5 (14.7)
Male	76 (73.8)	Range	6-120
Female	27 (26.2)	6-12	23 (22.3)
**Current marital status**		13-18	26 (25.2)
Single	37 (35.9)	19-24	28 (27.2)
Married	59 (57.3)	More than 24	26 (25.2)
Divorced	7 (6.8)		
Separated			
**Average monthly income after injury (Uganda shillings)**		**ASIA Impairment Scale (Completeness of injury)**	
None	64 (62.1)	Complete (Asia A)	23 (22.3)
<500,000	33 (32)	Incomplete (Asia B, C & D)	60 (58.3)
>=500,000	6 (5.8)	Asia E	20 (19.4)
**Employment status**		**Level of injury**	
Unemployed	75 (72.8)	Cervical	39 (37.9)
Employed	28 (27.2)	Lumbar	31 (30.1)
**Highest level of education**		Thoracic	33 (32)
Primary	51 (49.5)		
Ordinary			
secondary	38 (36.9)		
Higher secondary	6 (5.8)		
University degree	8 (7.8)		

Road traffic accidents accounted for most injuries (59.2%). The mean duration since injury was 20.5 months (SD 14.7, range 6- 120). Most participants (58.3%) had incomplete SCI while 84.5% had complications. Cervical spine injury was the most common in 37.9% of patients as shown in Table 1. Overall, most participants (61.2%) had a poor QOL; only 12.6% reported to have a good QOL (Table 2).

**Table 2:** Perceived QOL

Perceived QOL	Frequency (Percent)
Very Good	0 (0)
Good	13 (12.6)
Neither Good nor Poor	27 (26.2)
Poor	58 (56.3)
Very Poor	5 (4.9)

Marital status (P-value <0.013), employment status (P-value <0.001), average monthly income after injury (P-value <0.001) and injury severity significantly affected perceived QOL as shown in Table 3.

**Table 3:** Predictors of overall QOL

Characteristic	Mean (SD)	F-statistic	P-value
**Overall QOL**	150.5(48.9)		
**Age**		0.29	0.942
18-27	146.8 (50.9)		
28-37	154.4 (50.7)		
38-47	148.2 (44.8)		
48-57	148.5 (48.1)		
58-67	159.2 (53.4)		
68-77	106.0 (0.0)		
78-87	168.0 (62.2)		
**Sex**		0.07	0.798
Male	151.2 (49.8)		
Female	148.4 (47.2)		
**Current marital status**		4.58	0.013*
Single	141.9 (47.8)		
Married	160.7 (48.7)		
Divorced	109.9 (25.0)		
**Average monthly income after injury**		10.17	<0.001*
None	135.4 (47.1)		
<500,000	171.5 (41.7)		
>=500,000	195.7 (39.8)		
**Employment status**		24.75	<0.001*
Unemployed	137.3 (45.4)		
Employed	185.9 (40.2)		
**Mechanism of injury**		0.31	0.735
Fall from a height	160.0 (59.8)		
Road traffic crush	150.0 (47.0)		
Other (includes assault & gunshot injury)	147.2 (49.1)		
**Presence of complications**			<0.001*
Symptom absent	196.6 (31.8)		
Symptom present	142.0 (46.9)		
**Duration of Injury (Months)**		2.03	0.115
6-12	151.3 (47.6)		
13-18	144.7 (38.2)		
19-24	167.9 (56.7)		
Above 24	136.9 (47.7)		
**ASIA Impairment Scale**		21.22	<0.001*
Complete (Asia A)	125.7 (33.2)		
Incomplete (Asia B, C & D)	142.6 (45.3)		
Asia E	202.9 (37.1)		*p< 0.05
**Level of injury**		1.43	0.243
Cervical	144.7 (47.9)		
Lumbar	162.9 (46.3)		
Thoracic	145.8 (51.8)		

Employment status was significantly associated with physical health (Adj. β, CI 2.1 – 14.9, p= 0.009) and psychological wellbeing (Adj. β 11.9, CI 2.8 – 20.9, p= 0.011) as shown in Table 4. Presence of complications had a negative influence on psychological wellbeing (Adj. B -8.5, CI -16.1- -0.9, p= 0.03) and social relationships (Adj. β -11.9, CI -19.1- -4.6, p= 0.002). ASIA E impairment was significantly associated with psychological wellbeing ((Adj. β 13.6, CI 5.3 – 21.9, p= 0.02), social relationships (Adj. β 9.3, CI 0.1 – 18.4, p= 0.047) and the environment domains (Adj. β 12, CI 4.9 – 19.5, p= 0.001). Married participants had better social relationships (Adj. β 13.5, CI 8.1- 13.9, p< 0.001). The influence of factors on QOL domains is summarized in Table 4.

**Table 4:** Multivariate analysis of factors influencing QOL

DOMAIN	Characteristic	Adjusted β	95% CI	P-value
**Physical health**	**Employment status**			
	Unemployed	0		
	Employed	8.5	2.1 - 14.9	0.009*
	**Presence of complications**			
	Symptom absent	0		
	Symptom present	-5.9	-12.3 - 0.4	0.066
	**ASIA Impairment Scale**			
	Complete (ASIA A)	0		
	Incomplete (ASIA B, C & D)	-2.7	-8.9 - 3.4	0.378
**Psychological well-being**	**Employment status**	11.9	2.8 - 20.9	0.011*
	Unemployed	0		
	Employed	8.3	2.6 - 13.9	0.005*
	**Presence of complications**			
	Symptom absent	0		
	Symptom present	-8.5	-16.1 - -0.9	0.029*
	**ASIA Impairment Scale**			
	Complete (ASIA A)	0		
	Incomplete (ASIA B, C & D)	1.2	-3.7- 6.2	0.622
	ASIA E	13.6	5.3 - 21.9	0.002*
**Social Relationships**	**Marital status**			
	Single	0		
	Married	13.5	8.1 - 19.0	<0.001*
	Divorced	-4.1	-12.7 - 4.6	0.353
	**Presence of complications**			
	Symptom absent	0		
	Symptom present	-11.9	-19.1 - -4.6	0.002*
	**Asia Impairment Scale**			
	Complete (ASIA A)	0		
	Incomplete (ASIA B, C &D)	3.8	-2.8 - 10.5	0.253
	ASIA E	9.3	0.1 - 18.4	0.047*
**Environment**	**Highest level of education**			
	Primary	0		
	Ordinary secondary	4.8	0.3 - 9.2	0.037*
	Higher secondary	7.0	-1.3 - 15.3	0.097
	University degree	4.1	-4.7- 13.0	0.358
	**Average monthly income after injury**			
	None	0		
	<500,000	5.9	1.4 - 10.3	0.010*
	>=500,000	9.0	-2.3 - 20.4	0.117
	**ASIA Impairment Scale**			
	Complete (ASIA A)	0		
	Incomplete (ASIA B, C & D)	2.7	-2.5 - 7.9	0.304
	ASIA E	12.2	4.9 - 19.5	0.001*

* p< 0.05

On multivariate analysis, being employed (p= 0.02), presence of symptoms (p= 0.03), and ASIA E impairment (p= 0.003) remained significant (Table 5).

**Table 5:** Multivariate analysis of predictors of overall QOL

DOMAIN	Characteristic	Adjusted β	95% CI	P-value
**Overall QOL**	**Employment status**			
	Unemployed	0		
	Employed	26.4	5.3, 47.5	0.015*
	**Presence of complications**			
	Symptom absent	0		
	Symptom present	-28.0	-53.0, -3.0	0.028*
	**ASIA Impairment Scale**			
	Complete (ASIA A)	0		
	Incomplete (ASIA B, C & D)	7.0	-10.9, 25.0	0.437
	ASIA E	47.4	16.4, 78.5	0.003*

* p< 0.05

Individuals with lumbar spine involvement and those with ASIA E injuries had better FIM scores of 113.1±8.9 and 99.9±15.3 respectively (Table 6).

**Table 6:** Association between Functional Independence Measure score, level and severity of injury

Characteristic	Mean FIM score	SD	P-value
**ASIA Impairment Scale**			<0.001*
Complete spinal cord injury (ASIA A)	79.0	14.7	
Incomplete spinal cord injury (ASIA B, C & D)	90.2	16.5	
Asia E	113.1	8.9	

**Level of injury**			0.002*
Cervical	84.5	20.2	
Thoracic	93.9	16.3	
Lumbar	99.9	15.3	

* p< 0.05

## Discussion

Traumatic SCI are devastating and disruptive to an individual's life. With the increasing long-term survival after SCI, health related QOL has become crucial in the rehabilitation process. Therefore, enhancing a high level of QOL has become the main goal during rehabilitation 27. The self-reported overall QOL among patients with TSCI in this study was generally poor. Presence of complications had a negative influence on the overall QOL whereas being married, having employment and ASIA E (mild SCI) had positive influence. Injury completeness did not significantly influence QOL. Just like in most sub-Saharan Africa, motor vehicle accidents, fall from heights and assault were the major causes of injury 7-12.

The self-reported QOL in the vast majority of participants was rated as poor because a majority had no functional independence and already had complications. Functional independence was assessed using the FIM and this gave us a crude idea on the participants' recovery process six months after discharge from hospital. There was significant decrease in the FIM from the cervical spine to the lumbar spine. The same trend was also observed with overall QOL. Consistent with our findings several authors have reported lower QOL in patients with higher level SCI 13, 27, 28. This is also consistent with Barbetta et al. who used FIM to discriminate functional ability in different spinal of levels 23. It is important to note that the FIM is of prognostic value in SCI rehabilitation and is essential in assessing for functional recovery and independence 29-33. Improvement in FIM has been associated with reduced burden and longer survival in SCI patients 33. These results suggest that level of injury is a major factor influencing QOL due to its potential effect on an individual's functional independence and the ability to perform activities of daily living.

The mean overall QOL score in this study was lower in comparison to other parts of the world 11, 34-37; possibly due to the inadequate care of TSCI in the Uganda 38. Patients living with SCI have complex needs that require a multidisciplinary approach to management; however, the care of these patients in Uganda is sub-optimal and this impacts on their QOL. Uganda as a country, does not have even a single specialized institution or program dedicated to the rehabilitation and re-integration of SCI patients back in the community 39. About 85% of our participants already had complications, and this significantly influenced their QOL. Most patients with SCI are discharged from hospital without any comprehensive follow up or rehabilitation program 39. Oftentimes they are referred to peripheral health facilities in their home districts for follow up and rehabilitation however, most of these facilities are poorly equipped, do not have physiotherapists/occupational therapists, and community support is inadequate 39, 40. Further, the available physical and occupational therapists have limited experience in managing these patients; and this predisposes them to complications and frequent hospitalization 39. A previous study in Uganda among patients with long-term SCI reported inadequate home care, lack of support for family caregivers and lack of assistive devices like wheelchairs and walking aids 39. There is therefore need for the establishment of a comprehensive program for the care of people living with SCI in Uganda if their QOL is to significantly improve.

Another factor that could contribute to the poor overall QOL is the sub-optimal emergency care SCI patients receive in Uganda. The treatment outcome of TSCI is majorly impacted by the quality and timeliness of medical care immediately after sustaining the injury 41-44. The management of SCI requires special care right from the site of accident. Unfortunately, there is no public ambulance system in Uganda, most patients are just loaded onto pickup trucks by lay first responders 45 and untrained police officers for transfer to hospital; and in the process, there could be aggravation of any SCI. In addition, the Spine Unit at the national referral hospital is the only public referral unit in the country that specializes in surgical care of spine ailments and injuries. Spine surgery is key in the management of SCI; it improves neurological recovery and reduces the duration of hospitalization 43, 44, 46. Spine surgery also contributes to stabilization of the vertebral column and facilitates sitting, transfer, ambulation and rehabilitation. However, the Spine Unit does not have adequate capacity to provide spine stabilization surgery to all those who need it. Surgical stabilization of the spine requires expensive implants, that are usually in short supply. The Spine Unit depends on Western donations for surgical implants, with minimal contribution from government 47. Once the stock of donated implants is depleted, patients have to purchase these very expensive implants that can cost up to 3000$ 47, in a country with a gross domestic product per capita of 777$ 48. Scarce resources in the Ugandan health system therefore leaves many injured patients untreated or sub-optimally treated causing significant long-term disability 49.

A greater majority of participants (75%) in this study were unemployed, and 94% had a monthly income of less than 500,000 Uganda Shillings (142 US Dollars). Being employed and earning less than 500,000 Uganda Shillings were predictors of overall QOL. Employment positively influenced the physical health (P=0.009) and psychological well-being (P=0.005) domains of QOL. A sufficient household income is important for a person's well-being. Studies show that people living with SCI who are employed report a better QOL 34, 50-53. A 10-year longitudinal study in the United States of America reported that patients with SCI with low household income had poorer subjective well-being than those with high income, and experienced more health problems 53. Gainful employment facilitates independent living and functioning, socialization, boosts self-esteem, and financial self-sufficiency 54. Functional dependence and disability restrict an individual's engagement in income generating activities and causes financial strain that may contribute to a poor QOL 55. The majority of the Ugandan population works in the informal sector, surviving on subsistence farming where people use traditional methods of farming, that majorly require manual labour. Therefore, loss of the ability to perform manual tasks not only affects activities of daily living but also loss of employment. This in turn has a strong bearing on their social relationships and psychological well-being.

All factors in this study were significantly associated with psychological wellbeing and social relationships. Furthermore, married participants reported a better QOL than the single or divorced/separated individuals (p< 0.001). Married people living with SCI have been reported to have better satisfaction with life and family support 56, 57. They benefit physically and emotionally from their spouses and easily adapt to their new life situation. Spousal support also offers the much-needed social support and facilitates societal integration 58. On the other hand, caring for a patient living with SCI places a heavy economic, physical and emotional burden on the primary caregivers 59. Literature shows that SCI significantly impacts the QOL of caregivers; many of them experience challenges including depression, anxiety, physical symptoms associated with manual patient handling, and reduced satisfaction with life 60. Therefore, there is need for a multidisciplinary approach to caring for SCI patients that should include among other things, focused interventions to support patients and caregivers with special emphasis on patient/caregiver education and access to support services 60.

Several factors in this study were not significant predictors of QOL. Younger age has been reported as a predictor of QOL 61-64 however in this study the relationship with QOL was not significant. The correlation between duration of injury and QOL in this study was not significant. The correlation between these two factors in the literature is inconclusive. Whereas some studies have reported that shorter duration of injury positively influences QOL 34, others have reported a negative influence 62. Other scholars have reported longer time since injury as being associated with better QOL 27, 34. On the other hand, Sabour et al^28^ reported no relationship between time since injury and QOL. Therefore, there is a need for longitudinal studies to further investigate the relationship between duration of injury and QOL in SCI. Consistent with our findings, the insignificant effect of injury completeness on QOL has been previously reported by several authors 27, 65, 66.

Higher level of education did not significantly influence overall QOL; and this may due to the very few participants (14/103) who had higher and university level education.

In order to improve the QOL of individuals with SCI, there is need for programs that help reintegrate them in community upon discharge from hospital. However, this is often hindered by the lack of effective policies on community integration for people with disabilities and the negative community perceptions that view these people as worthless and burdensome 67, 68. Many of these patients find it difficult to adapt to their new life situation, suffer stigma, discrimination and usually develop mental health problems 55, 69-71. Mitigation of these problems requires a multi-disciplinary approach. Affected individuals and their families need adequate health education and counselling to come to terms and live positively with their injuries. There is also need for improved community accessibility, public sensitization and awareness campaigns to promote community integration and improve societal understanding of SCI. This study is the first to quantitatively document the QOL of people living with SCI in Uganda and used assessment tools that have been widely used and has shown cross-cultural validity 72-75.

There were some study limitations. The study did not conduct an in-depth exploration of the various complications participants had and how these affected their QOL. The sample size was relatively small and participants were consecutively recruited from a single institution hence the results may not be generalizable to the entire country. The cross-cultural content validity of the WHO-QOL BREF questionnaire was also assumed in this study. The FIM was assessed at a one point in time however it is of better utility when done serially with time. Therefore, there is a need for a longitudinal study to investigate the utility of the FIM our setting.

## Conclusion

The overall self-reported QOL among patients with TSCI was generally poor. Level of injury is a major factor influencing QOL. Patients who were either married or had spouses, and the those that were employed had a significantly better QOL. Injury completeness had no significant influence on QOL. There is need for the establishment of a comprehensive rehabilitation program to strengthen capacity for the care of people living with SCI in Uganda.
